# Adolescent bulimia nervosa (BN): a new therapeutic frontier

**DOI:** 10.1186/2050-2974-2-S1-O6

**Published:** 2014-11-24

**Authors:** Kim Hurst, Shelly Read, Tiegan Holtham

**Affiliations:** 1 Eating Disorder Program, Gold Coast CYMHS, Robina, Australia; 2 CYMHS Gold Coast, Robina, Australia

## 

Enhanced Cognitive Behaviour Therapy (CBT-E) continues to gather evidence as the first line of treatment for Bulimia Nervosa (BN) in adulthood. However, there is limited evidence for empirically validated therapies for children and adolescents, despite the serious impact of medical complications during adolescence.

In recent years, investigation into the effectiveness of Family Based Treatment (FBT) for BN has been instigated. It has been proposed that FBT may be particularly helpful for BN, by combating the secretive and shameful nature of BN, and increasing adolescent collaboration. The following case study contrasts the delivery of FBT to two adolescents presenting with BN; one is augmented with CBT-E.

In both cases, the adolescents achieved remission from BN symptoms, with a cessation of bingeing and/or compensatory behaviours at the end of treatment. Parents and adolescents reported feeling as though FBT provided a platform to work together and view BN as a family issue, rather than leaving the onus on the young person to recover independently. Both families reported higher levels of cohesion at the end of treatment, and parents reported feeling more empowered to help their offspring. The application of specific CBT-E strategies was also reported as integral in achieving recovery. Further research in this area is indicated.

This abstract was presented in the **Treatment in Community and Inpatient Settings** stream of the 2014 ANZAED Conference.

